# Implementation of cancer treatment during pregnancy in daily practice: the important role of perinatologists

**DOI:** 10.18632/oncotarget.25841

**Published:** 2018-08-07

**Authors:** Koen F. de Geus, Charlotte Maggen, Jorine de Haan, Frédéric Amant

**Affiliations:** Frédéric Amant: Center for Gynaecologic Oncology Amsterdam, Amsterdam University Medical Centers, Amsterdam, The Netherlands; Department of Obstetrics and Gynaecology, University Hospitals Leuven, Leuven, Belgium; Center for Gynaecologic Oncology Amsterdam, Antoni van Leeuwenhoek-Netherlands Cancer Institute, Amsterdam, The Netherlands

**Keywords:** pregnancy, perinatology, neonatal outcome, cancer treatment

Founded in 2005 at the KU Leuven, Belgium, the International Network on Cancer, Infertility and Pregnancy (INCIP), set its ambitious goal to improve evidence on all aspects concerning cancer during pregnancy. In the past two decades, the network has grown, now consisting of over 95 member medical centers from over 30 different countries worldwide.

Currently, incidence of cancer in pregnancy is estimated at one in 1000 pregnancies [1, 2]. With maternal age at first childbirth increasing [3], we anticipate this number to increase in the coming years. To provide future patients with comprehensive and evidence-based information on both oncological and obstetric management and outcome, INCIP initiated various research projects to study a broad range of issues related to cancer in pregnancy; from analysis of the effects of oncological treatment on maternal and fetal outcome to follow-up of children that were antenatally exposed to these treatments and assessment of psychological impact on patients and their partners diagnosed with cancer in pregnancy.

Recently, INCIP published results of an ongoing 20-year cohort study on both the oncological and obstetric management and outcomes of 1170 patients [4]. This study included patients both retrospectively (before 2005) and prospectively (from 2005 onward) who were diagnosed with primary invasive cancer during pregnancy, analyzed the advancement of oncological management during pregnancy in the past decades, and the observed obstetric and neonatal outcomes. Breast cancer was the most common cancer type during pregnancy (39.5%), followed by gynecological (13% cervical cancer and 7% ovarian cancer) and hematological (10% lymphoma and 6% leukemia) cancers. Most patients were diagnosed in their second trimester, with locally-staged disease. The majority of patients were multiparous at time of diagnosis.

It was found that two-thirds of all patients received some sort of oncological treatment during pregnancy, with the likelihood of receiving treatment during pregnancy increasing with 10% every five years in the last twenty years (RR 1.10; 95% CI 1.05-1.15). Simultaneously, every five years more live births (RR 1.04; 1.01-1.06) and fewer iatrogenic preterm births (RR 0.91; 0.84-0.98) were observed. Twenty-one percent of the neonates were born small for gestational age (SGA), defined as a birth weight below the 10^th^ percentile. Multivariate analysis showed increased risks on SGA after antenatal chemotherapy exposure consisting in general (p<0.0001), specifically after exposure to platinum derivatives or taxanes (OR 3.12 (95% CI 1.45-6.70) and 2.07 (95% CI 1.11-3.86), respectively). Also, the frequency of SGA rose for each 5-year study period, reflecting the increasing tendency to treat during pregnancy.

With preterm birth being associated with reduced cognitive development [5], the observed decrease in incidence of iatrogenic prematurity is considered a positive advancement. However, the potential negative effects of children born SGA should not be underestimated, as perinatal mortality and morbidity, and cardiovascular and metabolic disorders later in life are more common in these children [6].

Despite the extensive network and careful efforts of all participating specialists involved in registering patients into the INCIP registry, the partial retrospective study design entails the risk of selection bias. Also, as included patients were diagnosed in varying countries and at various time points (diagnosis between 1996 and 2016), factors regarding diagnosis and treatment could greatly vary between patients. Missing data on fetal growth during pregnancy prevent to identify intra-uterine growth restricted fetuses [7], which definition is based on different criteria (declining growth (crossing centiles more than 2 quartiles) in sequential ultrasound measurements, Doppler measurements (Pulsatility Index of the umbilical artery >95^th^ percentile), abdominal circumference <10^th^ percentile) compared to SGA. Cytotoxic drugs during pregnancy have the potential to affect placental function. As a result of impaired placental function, growth restricted fetuses fail to reach their growth potential, but not necessarily have a birth weight below the 10^th^ percentile. INCIP now aims to collect more cases with detailed information on fetal growth to explore the effects of different cancer treatments on fetal outcome.

Nevertheless, the study’s reported outcome measures were based on one of the largest cohorts observed to date. As cancer in pregnancy is a rare phenomenon, collaboration in international networks is vital to the expansion of evidence. Additional research is warranted to further investigate the effect of cancer and cancer therapeutics on placental physiology, management of rare histological subtypes and uncommon or newer treatment options. The study group continues to register young women with a cancer diagnosis in association with pregnancy. Currently, 1625 patients are registered in the online database, whose access is restricted to INCIP members (June 2018, Figure 1).

**Figure 1:**
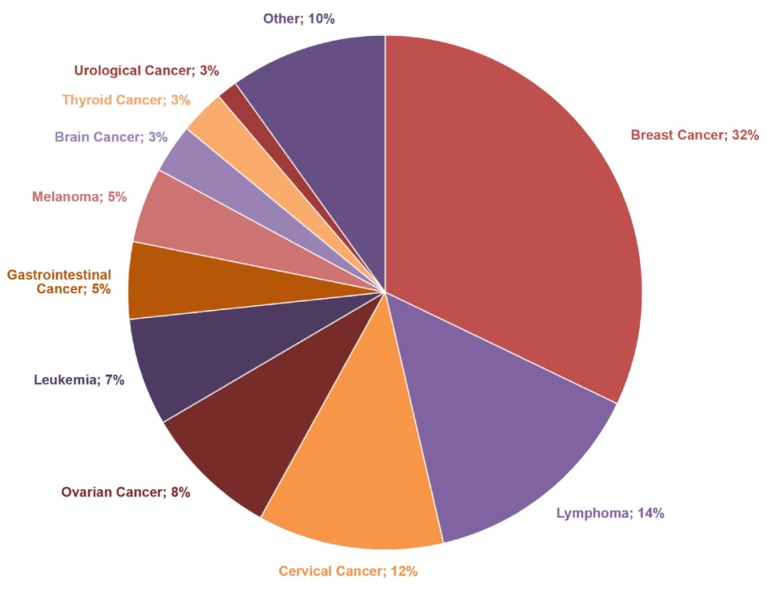
Distribution of cancers diagnosed during pregnancy in INCIP registry; 1625 cases registered in June 2018

We speculate that with ongoing and future research we could achieve further knowledge on cancer treatment for pregnant women, making evidence-based and individualized decisions to achieve the best possible outcomes for both mother and child. With the ongoing expansion of our network and based on previously published data and clinical questions, we aim to extend our research to unravel the exact effects of chemotherapy on placental function and evaluate pharmacokinetics and distribution of systemic treatment modalities in the pregnant population. To date, retrospective studies reveal no significant impact of pregnancy on maternal outcome, although it is speculated that pregnancy may potentially alter pharmacokinetics and efficacy of cancer treatment [8, 9]. More detailed analysis of long-term follow-up of patients that received cancer treatment during pregnancy is needed, taking into account possible confounders of prognosis. Moreover, we aim to gain insight in impact and psychological support for women diagnosed with cancer during pregnancy and to extend research into effects on lactation and neonatal immunity when exposed to chemotherapy in utero.

We invite all specialists with a specific interest in cancer in pregnancy worldwide to participate in INCIP, and to help construct a robust foundation of evidence-based medicine for women faced with a grim diagnosis while expecting a child.

For INCIP registry, see cancerinpregnancy.org. To register for INCIP, see https://incipregistration.be.

## References

[1] Lee YY (2012). BJOG.

[2] Parazzini F (2017). Int J Gynecol Cancer.

[3] Eurostat (2018). http://ec.europa.eu/eurostat/web/products-datasets/-/tps00017.

[4] de Haan J (2018). Lancet Oncol.

[5] Amant F (2015). N Engl J Med.

[6] Madden JV (2018). Am J Obstet Gynecol.

[7] Gordijn SJ (2016). Ultrasound Obstet Gynecol.

[8] Stensheim H (2009). J Clin Oncol.

[9] Amant F (2013). J Clin Oncol.

